# High-incidence of PTEN mutations in Chinese patients with primary small cell carcinoma of the esophagus

**DOI:** 10.1186/1471-2407-14-19

**Published:** 2014-01-14

**Authors:** Zhimin Zhang, Hualiang Xiao, Fei Xie, Hui Zhang, Chuan Chen, He Xiao, Zhenzhou Yang, Dong Wang, Zengpeng Li, Ge Wang

**Affiliations:** 1 Cancer Center, Institute of Surgical Research, Daping Hospital, Third Military Medical University, Chongqing 400042, China; 2 Department of Oncology, Wuhan General Hospital of Guangzhou Command, People’s Liberation Army, Wuhan, Hubei 430070, China; 3 Department of pathology, Daping Hospital, Third Military Medical University, Chongqing 400042, China

**Keywords:** Primary small cell carcinoma of the esophagus, PTEN, EGFR, KRAS, PIK3CA, Mutation

## Abstract

**Background:**

Primary small cell carcinoma of the esophagus (PSCCE) is a rare and aggressive tumor with poor prognosis. The aim of this study was to investigate the existence of EGFR, KRAS, PIK3CA and PTEN mutations in PSCCE.

**Methods:**

Clinical–pathological data and paraffin-embedded specimens were collected from 38 patients. Exons 18 to 21 of EGFR, KRAS and PIK3CA status were analyzed by real-time PCR based on ARMS and Scorpion technology in all patients, and the PTEN gene was also screened using real-time PCR and high-resolution melting curve analysis (HRMA).

**Results:**

Only 1 (2.63%) out of 38 patients had EGFR mutations in L858R missense, and KRAS and PIK3CA were not found in the mutational spot in all patients. However, PTEN mutations presented in 14 (36.84%) out of 38 patients, including exon 5 coding for PTEN missense mutation (*n* =4, 10.53%), exon 6 (*n* =7, 18.42%), concurrent exon 5 and exon 6 (*n* =2, 5.26%), and exon 8 (*n* =1, 2.63%). Concurrent mutations of these genes were not detected in all samples. No statistically significant associations were found between the clinicopathological features and the mutation status of PTEN.

**Conclusions:**

The incidence of PTEN mutations in Chinese patients with PSCCE was higher than that of previous reports in other histological subtypes of esophageal cancer.

## Background

Primary small cell carcinoma of the esophagus (PSCCE) is a specific histological type of esophageal malignancy and is a rare, aggressive disease with a high metastatic rate and poor outcome. The incidence of PSCCE is reported to be 1–1.5% of all esophageal malignancies [1] and from 0.05 to 2.4% in western populations [2], 7.6% in Chinese literature [2,3]. Several treatment options are available, including surgery, chemotherapy, radiotherapy and concurrent chemo-radiotherapy, but the prognosis remains poor. Hence, it is urgent to explore novel therapeutic modalities for patients with PSCCE.

Molecular targeted therapy is one of the new modalities that have emerged in the past decade. An epidermal growth factor receptor (EGFR) has been validated as a promising therapeutic target for cancer. It has been reported that EGFR expression is higher in esophageal cancer cells than in corresponding normal tissue and EGFR mutations have always been found although the incidence is low [4-15]. And whether or not it may be potentially useful targets of therapy for esophageal cancer remains unclear.

V-Ki-ras2 Kirsten rat sarcoma viral oncogene homolog (KRAS) is a critical downstream effector of the EGFR pathway. KRAS can harbor oncogenic mutations that yield a constitutively active protein [16]. Recently studies have indicated that the presence of mutant KRAS is favorable to one of the high-risk factors implicated in esophageal squamous cell carcinoma (ESCC) development [17-21]. Mutant Phosphatidylinositol 3-kinase CA (PIK3CA) stimulates the AKT pathway and promotes cell growth in several cancers, including ESCC and Non-small cell lung cancer (NSCLC) being associated in these cases with poor prognosis [22]. Furthermore, PIK3CA mutations were always found in esophageal cancer [23,24] and further functional analyses of the mutations are warranted to determine whether or not they may be potentially useful targets of therapy for esophageal cancer [25]. Phosphatase and tensin homolog deleted on chromosome 10 (PTEN) mutation is a frequent event in endometrial cancers. Recent reports have demonstrateded that the presence of PTEN mutation is highly predictive in glycogenic acanthosis of the esophagus, and there are mutations in the PTEN gene of the ESCC cells and that the wild type PTEN gene has important effects on the ESCC cells in vitro and in vivo [26,27]. Whatever, these data suggest that PTEN could be another target gene in esophageal cancer treatment.

Mutations in KRAS, PIK3CA and PTEN genes have recently emerged as the potential predictive factors of low/absent response to EGFR-targeted therapy. Given that currently there is a lack of data on gene mutations associated with EGFR, a potential target for PSCCE, except a few case reports which lacks detailed description of the type of esophageal cancer investigated, and the distribution of these genes mutations in PSCCE still remains uncertain, we were motivated to conduct this study. The present study, which to our knowledge is the first in the world on this area, will help to clarify the issues.

## Methods

### Clinical specimens

**Figure 1:**
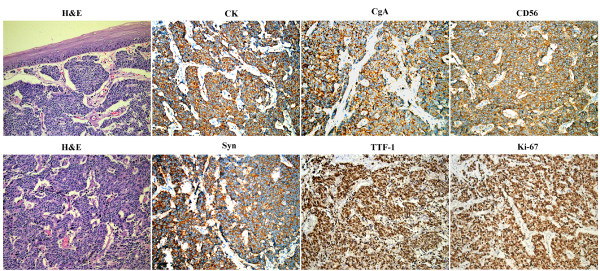
**A representative case of primary small cell carcinoma of the esophagus.** Biopsy materials were shown in the histopathologic examination, and tumoral cells in the sample indicated CK, CgA, CD56, Syn, TTF-1 and Ki-67 with a positive immunoreactivity by immunohistochemical examination. (Magnification, ×200).

38 samples of cancer tissues were obtained from PSCCE patients who underwent endoscopic evaluation with biopsy and esophagectomy at Daping Hospital, Third Military Medical University between October 2007 and June 2012. The age of the patients ranged from 42 to 76 years (median 61.3 years). 31 (81.58%) patients were male and the rest 7(18.42%) were female. The patients were treatment-naive prior to the study. Esophageal biopsies were obtained via endoscopy from the 38 patients and histopathology performed. The cells, forming neoplastic formation in which mitotic figures and intensive squeezed artefacts were found, were round or oval-shaped and having the granular chromatin. The cytoplasm was narrow, and nuclei appeared in different shapes (Figure 1). Immunohistochemical (IHC) assay demonstrated that tumoral cells in over 95% of the 38 samples presenting chromogranin A (CgA), Ki-67, cytokeratin (CK), and synaptophysin (Syn) with a positive immunoreactivity. Immunoreactivity together with thyroid transcription factor-1 (TTF-1) and CD56 were observed positive in about 45% to 65%. All the cases were reported as PSCCE by three independent pathologists with knowledge of clinical data, cytological and IHC examination. The study protocol was in accordance with the ethical guidelines of the 1995 Declaration of Helsinki and was approved by independent ethics committees at Daping Hospital, Third Military Medical University. Written informed consent was obtained from the patient for the publication of this report and any accompanying images.

### DNA isolation

Mutation analysis of these genes was performed by extraction of genomic DNA from formalin-fixed, paraffin-embedded tissue slides or sections with the use of the QIAamp DNA FFPE Tissue Kit (Qiagen), according to the manufacturer’s instruction. Tumor DNA was isolated from areas which were selected under light microscopic control by three senior pathologists and which containing at least 70% tumor cells in paraffin-embedded tumor samples.

### Mutation detection

#### EGFR, KRAS and PIK3CA mutation analysis

Mutational analysis was performed as described previously [17]. For EGFR mutations analysis, we used the EGFR Scorpions kit (DxS, Manchester, UK), which combines Scorpions amplification refractory mutation system (ARMS) and Scorpions technology, to detect mutations in Real-time Polymerase Chain Reaction (PCR) reactions. Mutant KRAS in exon 2 was detected using a validated KRAS mutation kit (DxS, Manchester, UK) that identifies seven somatic mutations located in codons 12 and 13 using allele-specific Real-Time PCR. PIK3CA mutations in exons 9 and 20 were detected using a validated PIK3CA mutation kit (DxS, Manchester, UK) that identifies three somatic mutations (H1047R, E542 and E545) by Real-Time PCR based on ARMS and Scorpion technology. All the analysis of these genes mutations were performed in an ABI Prism 7700 sequence detector (Applied Biosystems). SDS2.0 software (Applied Biosystems) was performed for data analysis according to the manufacturer’s instructions. Each sample was analyzed in triplicate or duplicate.

#### PTEN mutation analysis

PTEN mutations in exons 5, 6 and 8 were evaluated using a method previously published [28]. High-resolution melting analysis(HRMA) was performed on genomic DNA prepared from scraped paraffin slides. Two round PCR was done using 6 primer sets covering three exons of the PTEN gene. The following primer sets for Exon 5 were used: PTEN-F (forward) 5′ACC TGT TAA GTT TGT ATG CAA C3′, PTEN-R (reverse) 3′TCC AGG AAG AGG AAA GGA AA5′, Exon 6, PTEN-F 5′CAT AGC AAT TTA GTG AAA TAA CT3′; PTEN-R 3′GAT ATG GTT AAG AAA ACT GTT C5′, Exon 8, PTEN-F 5′CTC AGA TTG CCT TAT AAT AGT C3′; PTEN-R 3′TCA TGT TAC TGC TAC GTA AAC5′. All exons were amplified with the following PCR conditions: pretreatment at 94°C for 4 minutes, 35 cycles of amplification, and a single 10 minute final extension procedure. Each of these 35 cycles consisted of a denaturing step at 94°C for 1 minute, an annealing step of one minute (54°C for exons 5 and 8; and 53°C for exons 6), and an extension step at 72°C for 1 minute. After the final extension, an additional denaturation step at 95°C for 30 s was carried out. Subsequently, the PCR products were briefly centrifuged and were used directly for high-resolution melting using the LightScanner® instrument (Idaho Technology, Inc.). LightScanner® analytical software with Call-IT™ 2.0 (Idaho Technology, Inc.) was performed for data analysis according to the manufacturer’s instructions. All HRM assays were replicated two to three times for each sample.

#### Statistical analysis

All the data were processed using SPSS13.0 software. Chi-square (*X*^*2*^) test was performed to assess the significance of the association between PTEN mutations and other clinical pathologic characteristics, e.g. gender, age (<60 vs. ≥60), tumor location (upper vs. middle vs. lower third), and TNM stage (cI vs. cIIa-cIIb vs.cIII). All *P*-values < 0.05 were considered as statistically significant.

## Results

**Figure 2:**
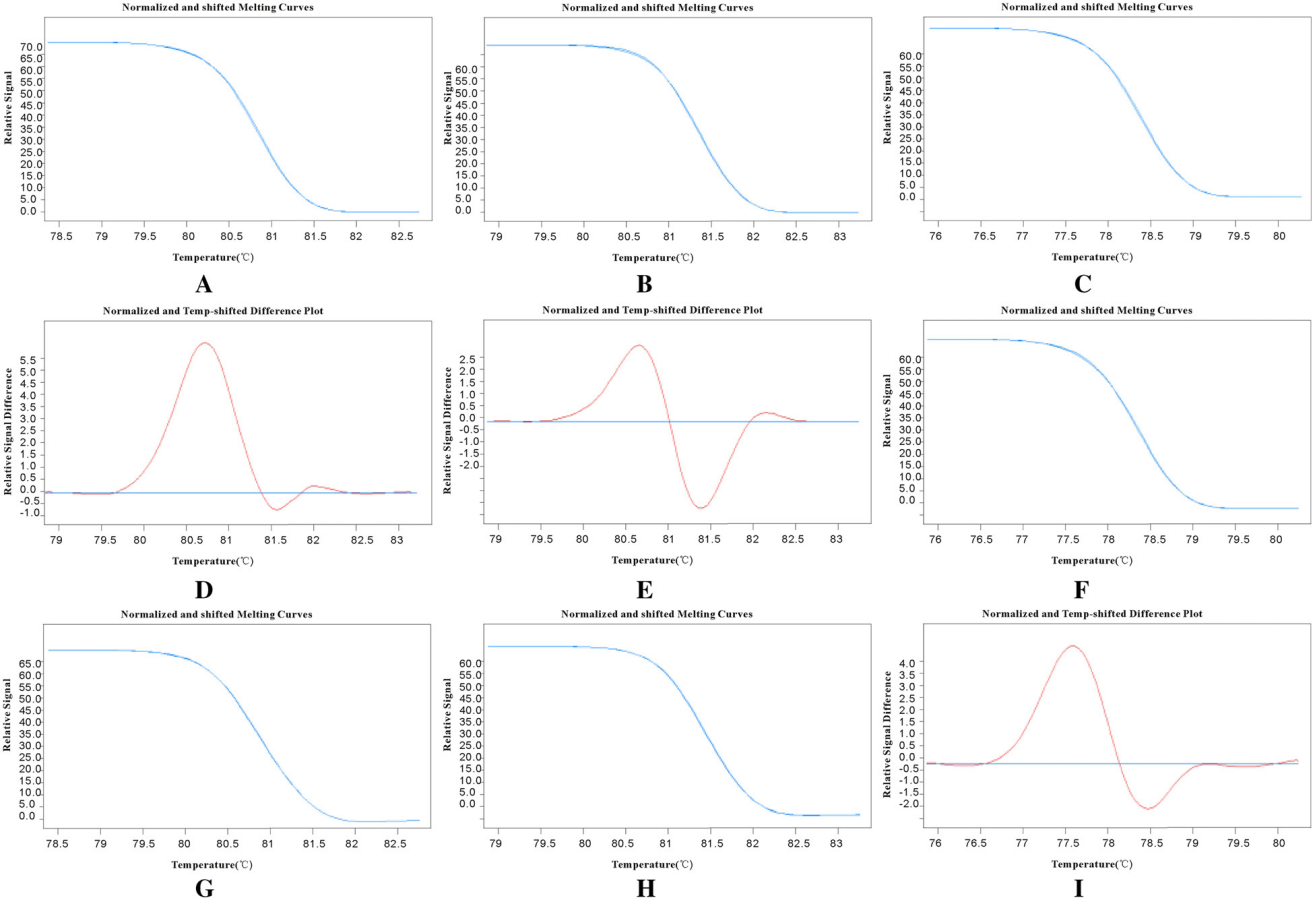
**Amplification plots for PTEN mutations in exon 5,6,8 using High-resolution melting analysis. (A,B,C)**. Amplification plots for a sample containing no mutated in PTEN gene. A, exon 5; B, exon 6; C, exon 8. **(D,E,F)**. Amplification plots for a sample containing mutated exon 5 and exon 6 in PTEN gene. D, exon 5; E, exon 6; F, exon 8. **(G,H,I)**. Amplification plots for a sample containing a mutated exon 8 in PTEN gene. G, exon 5; H, exon 6; I, exon 8. The Y-axis shows the relative fluorescence for a measurement of the change in fluorescent signal versus a passive reference signal, X-axis shows the temperature (°C). Each sample was analyzed in triplicate or duplicate.

The presence of EGFR, KRAS, PIK3CA and PTEN mutations in 38 patients were listed in Table 1. Each patient was represented only once, where for each type of material the information from all the patient’s samples was merged. An EGFR mutation was identified in 1(2.63%) of the 38 PSCCE patients, resulting in L858R missense mutation in exon 21. No mutation was found in exons 18,19 and 20. We either did not found KRAS mutations in codons 12, 13 and PIK3CA mutations in exon 9 (E542/E545) and exon 20 (H1047R) in all samples. PTEN mutations were detected in 14(36.84%) out of 38 patients (Figure 2), including exon 5 coding for PTEN missense mutation (*n* =4, 10.53%), exon 6 (*n* =7, 18.42%), concurrent exon 5 and exon 6 (*n* =2, 5.26%), and exon 8 (*n* =1, 2.63%). No concurrent mutations of these genes were detected in all samples. Moreover, there were no significant associations between PTEN mutations and clinical pathologic characteristics, e.g. gender, age, tumor location and TNM stage (Table 2).

**Table 1:** The frequency of EGFR, KRAS, PIK3CA and PTEN mutations according to different patterns (n = 38)

**Patterns of mutations**	**No. of cases (%)**
**EGFR**	
Exon 18 (G719S)	0
Exon 19 (DEL)	0
Exon 20	
T790M	0
INS	0
S7681	0
Exon 21	
L858R	1 (2.63)
L861Q	0
**KRAS**	
Codon 12	0
Codon 13	0
**PIK3CA**	
Exon 9 (E542/545)	0
Exon 20 (H1047R)	0
**PTEN**	
Exon 5	4 (10.53)
Exon 6	7 (18.42)
Exon 8	1 (2.63)
Exon 5 and Exon 6	2 (5.26)

2 patients also mutated in both Exon 5 and Exon 6 for PTEN.

**Table 2:** Correlations between PTEN mutations and clinical pathologic characteristics of all patients with PSCCE (n = 38)

**Characteristic**	**All patients**	**PTEN mutation**	**X**^**2**^	**P value**
	**No. of cases**	**No. of cases (%)**		
Gender				
Male	31	12 (38.71)	0.252	0.615
Female	7	2 (28.57)		
Age				
<60	16	7 (43.75)	0.567	0.452
≥60	22	7 (31.82)		
Tumor location				
Upper 1/3	4	1 (25)	0.634	0.728
Middle 1/3	23	8 (34.78)		
Lower 1/3	11	5 (45.45)		
TNM stage				
cI	3	1 (33.33)	0.866	0.648
cIIa-cIIb	17	5 (29.41)		
cIII	18	8 (44.44)		

TNM stage: The AJCC (6th edition) was used for staging. Abbreviations: c, clinical.

*P*-values < 0.05 were considered as statistically significant.

## Discussion

China is an endemic region for esophageal cancers. The incidence has been reported as165–200/100,000 in China, Japan and Eastern Turkey, while it is only 3/100,000 in Europe and USA [2]. Recently many published reports have demonstrated that EGFR mutations were detected in EC cell lines and patients with EC (Table 3). A phase II study of advanced EC treatment by gefitinib indicated that patients with ESCC had a higher disease control rate [7]. Another phase II trial using gefitinib in advanced EAC showed that gefitinib (500 mg/d) were an active and generally well-tolerated treatment for EAC [8]. However, whether similar results exist in patients with PSCCE remains unclear. To date, the mutation status of EGFR and EGFR related genes in patients with PSCCE have not been reported because of the rare incidence of the specific histological type of esophageal cancer worldwide. In fact, the reported incidence of PSCCE among all esophageal malignancies is higher in Chinese population than in Caucasians [2]. In this study, we found that only 2.63% of 38 patients with PSCCE carring EGFR mutations, consistent with data that reported in the previous studies on other histological types of EC [9,14,24], but significantly different from other reports (Table 3) . Possible reasons for the discrepancy are that ethnic differences in the distribution of the EGFR mutations in EC may exist, and the sensitivity of technique used for mutation detection differs. Furthermore, the only one patient with PSCCE identified for EGFR mutation was L858R missense mutation in exon 21, termed as gefitinib-associated mutations. This suggests the gefitinib-based small molecular target therapy possibly can be appropriately applied in treating PSCCE patients that harbor this specific mutation as well.

**Table 3:** Studies on EGFR, KRAS, PIK3CA and PTEN mutations in Esophageal cancer

**Gene**	**Reference**	**No.**	**Pathology**	**Exon examined**	**Mutated codon with amino acid**	**Mutation detated N (%)**
EGFR	Kwak et al. [4]	17	EAC	18-21	L858R, E746-A750del	2 (11.76)
	Guo et al. [6]	10	EAC	18-21	A734P	1 (10.00)
		57	ESCC		E872, G873, P753	3 (5.26)
		17	ESCC (cell line)		S768I (Exon20)	1 (5.88)
	Janmaat et al. [7]	26	NR	18-21	-	0
	Hanawa et al. [15]	40	ESCC	19,21	-	0
	Puhringer et al. [9]	105	BAC	19,21	K754	1 (0.95)
	Sudo et al. [5]	1	EAC (cell line)	18-21	2607G > A (Exon20)	1 (100)
		18	ESCC (cell line)		NR	2 (11.11)
		50	NR		G719D	1 (2.00)
	Mir et al. [14]	54	ESCC	18-21	codon 746–750, codon 753, codon 719 (Exon 19)	3 (5.56)
	Sunpaweravong et al. [13]	48	ESCC	19,21	-	0
	Kaneko et al. [12]	57	ESCC	18-21	codon 787 (Exon 20)	19 (33.33)
	Marx et al. [19]	20	BAC	18-21	-	0
	Liu et al. [11]	50	ESCC	18-21	G719X, L858R, Exon9 in-frame deletion	7 (14.00)
	Tasioudi et al. [20]	44	ESCC	18-21	-	0
	Wang et al. [10]	65	adenocarcinoma of esophagogastric junction	18-21	codon 787 2361G- > A (Exon20)	19 (29.23)
	Maeng et al. [23]	70	ESCC	NR	P733L	1 (1.43)
	Kato et al. [29]	107	ESCC	NR	del745-750	1 (0.93)
KRAS	Lord et al. [21]	23	EAC	2	Codon12	7 (30.43)
	Janmaat et al. [8]	23	NR	2	Codon12,13	2 (8.70)
	Lyronis et al. [18]	30	ESCC	2	Codon12	5 (16.67)
	Marx et al. [19]	20	BAC	2-3	NR	1 (5.00)
	Liu et al. [11]	50	ESCC	2	Codon12	6 (12.00)
	Tasioudi et al. [20]	44	ESCC	2	Codon12	1 (2.27)
PIK3CA	Phillips et al. [24]	35	ESCC	1-20	G1624A, G1633A, G1635A (Exon9) C1027T (Exon4)	4 (11.43)
		50	EAC		G1624A (Exon9)	3 (6.00)
		17	BAC		-	0
	Janmaat et al. [7]	24	NR	9,20	-	0
	Mori et al. [25]	88	ESCC	9,20	E545K,E545Q(Exon9)	2 (2.27)
		2	ESCC (cell line)		E545K, E545Q(Exon9)	2 (100)
	Akagi et al. [22]	52	ESCC	1,9,20	Exon9	4 (7.69)
	Maeng et al. [23]	70	ESCC	9,20	E545K, E542K (Exon9), H1047R, H1047L (Exon20)	9 (12.86)
PTEN	Hu et al. [28]	33	ESCC	1-9	802-29 T - > C (Exon7)	1 (3.03)
					492 + 8 T-deletion (Exon5)	33 (100)
	Hou et al. [26]	3	ESCC (cell line)	1-9	Exon2,5,6,8 in EC907 cells	3 (100)
					Exon5,8,9 in Eca cells	
					Exon6,8,9 in EC1 cells	

BAC, Barrett’s adenocarcinomas; EAC, esophageal adenocarcinoma; EC, esophageal carcinoma; EGFR, epidermal growth factor receptor; ESCC, esophageal squamous cell carcinoma; NR, not reported; KRAS, V-Ki-ras2 Kirsten rat sarcoma viral oncogene homolog; PIK3CA , Phosphatidylinositol 3-kinase CA; PTEN, Phosphatase and tensin homologue deleted on chromosome 10.

EGFR is a transmembrane tyrosine kinase receptor that, on ligand binding, triggers two main signaling pathways. These include the RAS-RAF-MAPK mainly involved in cell proliferation, and the PI3K/PTEN/AKT signaling pathway, mainly involved in cell survival and motility-invasion. In our study, KRAS mutations in codons 12 and 13 were not involved in PSCCE. Although published reports have shown the mutations of KRAS were always detected in the EC (Table 3), the incidence varied among different histological subtypes [29]. These findings indicated that KRAS mutations are a rare event in the carcinogenesis of PSCCE and tumorigenic effects of KRAS gene are histology specific in EC. In terms of therapeutic implications, this also suggests that PSCCE patients with mutated KRAS should gain little or no benefit from RAS-targeted therapy.

In addition to KRAS, the EGF receptor also activates the PI3K/PTEN/AKT signaling pathway. The latter can be oncogenically deregulated either by activating mutations in the PIK3CA or by inactivation of the PTEN phosphatase. The PIK3CA gene encodes the p110α catalytic subunit of PI3K that regulates the PI3K/AKT pathways, known to play a critical role in cancer onset and progression. A novel candidate tumor suppressor gene, PTEN gene, known as another effector of PI3K/PTEN/AKT pathway, is always lost by mutations, deletions or promoter methylation silencing at high frequency in many primary and metastatic human cancers, which are important mechanisms for cell cycle progression, survival, metabolism and migration. In this study, PIK3CA mutations in exons 9, 20 did not occur in PSCCE, but 36.84% (14 of 38 patients) of the PSCCE samples were found harboring PTEN mutations. These findings indicate that PIK3CA/PTEN/AKT pathway may be an important pathway for effect in response of EGFR targeted therapy in PSCCE, and the mainly target effector is not PIK3CA but PTEN.

PTEN mutations have been identified in numerous human malignancies, including brain, ovary and prostate cancers, but they are rarely seen in carcinomas arising from the head and neck region (including esophagus) [26-28]. Hu et al. reported a mutation incidence of only 3.03% in 33 ESCCs [28] and no mutations were detected in the hot spot exon 5 (Table 3). In contrast, a recently study reported by Hou et al. [26] found high-mutations incidence of PTEN in three cells of ESCC and that the elevated expression level of the wild type PTEN gene in ESCC cells may increase the sensitivity of the cancer cells to chemotherapeutic drugs (Table 3). Interestingly, 36.84% of the PSCCE samples were found harboring PTEN mutations, much higher than the incidence reported previously in ESCC and other tumors. Explanations for the difference include: (1) High-resolution melting (HRM) analysis might be more sensitive than direct sequencing. (2) Mutation of PTEN gene may be the most frequent molecular event in PSCCE. Although the significance of this mutation remains uncertain nowadays, it is likely to play a major role in the carcinogenesis of PSCCE. This is because the mutation is in the sequence encoding the putative phosphatase domain and the hot spots, including exon 5 (10.53%), exon 6 (18.42%), concurrent exon 5 and exon 6 (5.26%), and exon 8 (*n* =1, 2.63%). However, whether these mutations in the intron affects transcriptional or post-transcriptional modulation is still to be elucidated, and their relevance for EGFR targeted therapy in PSCCE have not been investigated thus far. Accordingly, further functional analyses of the PI3K/PTEN/AKT pathway in PSCCE are warranted to determine whether or not they may be potentially useful targets of therapy for PSCCE.

In the present study, we did not find any significant correlations between the clinicopathological features and the mutation status of PTEN (Table 2), which may be partly due to the relatively small sample size. Larger studies are needed to draw a firm conclusion on these issues.

Nevertheless, we invoke caution as there are some caveats involved in this study. First, the data presented here, such as treatment details, survival, and disease control are not enough to draw firm conclusions about whether the mutations of these genes can serve as a molecular classifier that correlates with TKIs responsiveness in PSCCE and, therefore, further studies involving larger studies will be required for an in-depth analysis. Next, our work, even though interested in providing evidence for a newly found high incidence of PTEN mutation in PSCCE, is rather preliminary at this stage and a detailed characterization of the molecular mechanisms involved is required further experimental data for better understanding the functional role and significance of PTEN mutation in PSCCE.

## Conclusions

Our study is the first report of mutational analysis of EGFR, KRAS, PIK3CA and PTEN in a number of patients with PSCCE. These results have indicated that a high-incidence of PTEN mutation other than EGFR, KRAS or PIK3CA mutations in PSCCE. This suggests that PTEN is a potential target for PSCCE in the future. Furthermore, EGFR mutations in PSCCE are rare but do exist, especially gefitinib associated mutations such as L858R, therefore gefitinib-based gene targeted therapy at EGFR but not KRAS and PIK3CA genes, probably should be included in this carcinoma treatment regimens for patients harboring L858R mutation.

## Abbreviations

PSCCE: Primary small cell carcinoma of the esophagus; NSCLC: Non-small cell lung cancer; EGFR: Epidermal growth factor receptor; KRAS: V-Ki-ras2 Kirsten rat sarcoma viral oncogene homolog; PIK3CA: Phosphatidylinositol 3-kinase CA; PTEN: Phosphatase and tensin homologue deleted on chromosome 10; ESCC: Esophageal squamous cell carcinoma; EAC: Esophageal adenocarcinoma; EC: Esophageal carcinoma; HRMA: High-resolution melting curve analysis; ARMS: Amplification refractory mutation system.

## Competing interest

The authors declare that they have no competing interests.

## Authors’ contributions

WG conception and design of research; ZZM carried out the molecular genetic studies and drafted the manuscript. XHL helped with sample acquisition and performed experiments. XF, CC and XH analyzed data, ZH, YZZ, WD and LZP conceived the study. ZZM and WG edited and revised manuscript; ZZM, XHL and WG approved final version of manuscript; All authors read and approved the final manuscript.

## Pre-publication history

The pre-publication history for this paper can be accessed here:

http://www.biomedcentral.com/1471-2407/14/19/prepub

## References

[B1] Pantvaidya GH Pramesh CS Deshpande MS Jambhekar NA Sharma S Deshpande RK Small cell carcinoma of the esophagus: the Tata Memorial Hospital experience Ann Thorac Surg 2002 74 6 1924 1927 10.1016/S0003-4975(02)04061-4 12643374

[B2] Yekeler E Koca T Vural S A rare cause of the cough: primary small cell carcinoma of esophagus-case report Case Rep Med 2012 2012 870783 22461794 10.1155/2012/870783 PMC3296277

[B3] Sun KL He J Cheng GY Chai LX Management of primary small cell carcinoma of the esophagus Chin Med J (Engl) 2007 120 5 355 358 17376302

[B4] Kwak EL Jankowski J Thayer SP Lauwers GY Brannigan BW Harris PL Okimoto RA Haserlat SM Driscoll DR Ferry D Muir B Settleman J Fuchs CS Kulke MH Ryan DP Clark JW Sgroi DC Haber DA Bell DW Epidermal growth factor receptor kinase domain mutations in esophageal and pancreatic adenocarcinomas Clin Cancer Res 2006 12 14 Pt1 4283 4287 16857803 10.1158/1078-0432.CCR-06-0189 PMC3807136

[B5] Sudo T Mimori K Nagahara H Utsunomiya T Fujita H Tanaka Y Shirouzu K Inoue H Mori M Identification of EGFR mutations in esophageal cancer Eur J Surg Oncol 2007 33 1 44 48 10.1016/j.ejso.2006.10.034 17142003

[B6] Guo M Liu S Lu F Gefitinib-sensitizing mutations in esophageal carcinoma N Engl J Med 2006 354 2193 2194 10.1056/NEJMc052698 16707764

[B7] Janmaat ML Gallegos-Ruiz MI Rodriguez JA Meijer GA Vervenne WL Richel DJ Van Groeningen C Giaccone G Predictive factors for outcome in a phase II study of gefitinib in second-line treatment of advanced esophageal cancer patients J Clin Oncol 2006 24 1612 1619 10.1200/JCO.2005.03.4900 16575012

[B8] Ferry DR Anderson M Beddard K Tomlinson S Atherfold P Obszynska J Harrison R Jankowski J A phase II study of Gefitinib Monotherapy in advanced esophageal adenocarcinoma: evidence of gene expression, cellular, and clinical response Clin Cancer Res 2007 13 19 5869 5875 10.1158/1078-0432.CCR-06-1970 17908981

[B9] Puhringer-Oppermann FA Stein HJ Sarbia M Lack of EGFR gene mutations in exons 19 and 21 in esophageal (Barrett’s) adenocarcinomas Dis Esophagus 2007 20 9 11 10.1111/j.1442-2050.2007.00630.x 17227303

[B10] Wang WP Wang KN Gao Q Chen LQ Lack of EGFR mutations benefiting gefitinib treatment in adenocarcinoma of esophagogastric junction World J Surg Oncol 2012 17 10 14 10.1186/1477-7819-10-14 PMC3278359 22252115

[B11] Liu QW Fu JH Luo KJ Yang HX Wang JY Hu Y Yang H Bella E Identification of EGFR and KRAS mutations in Chinese patients with esophageal squamous cell carcinoma Dis Esophagus 2011 24 374 380 10.1111/j.1442-2050.2010.01155.x 21615826

[B12] Kaneko K Kumekawa Y Makino R Nozawa H Hirayama Y Kogo M Konishi K Katagiri A Kubota Y Muramoto T Kushima M Ohmori T Oyama T Kagawa N Ohtsu A Imawari M EGFR gene alterations as a prognostic biomarker in advanced esophageal squamous cell carcinoma Front Biosci 2010 15 65 72 10.2741/3607 20036807

[B13] Sunpaweravong P Suwiwat S Sunpaweravong S Puttawibul P Mitarnun W Correlation of epidermal growth factor receptor mutation, immunohistochemistry, and fluorescence in situ hybridization in esophageal squamous cell carcinoma J Med Assoc Thai 2009 92 9 1136 1142 19772171

[B14] Mir MM Dar NA Salam I Shah ZA Mutations in epidermal growth factor receptor gene in esophageal squamous cell carcinoma patients in Kashmir- a high incidence area of India Int J Health Sci 2008 2 2 17 25 PMC3068730 21475485

[B15] Hanawa M Suzuki S Dobashi Y Yamane T Kono K Enomoto N Ooi A EGFR protein overexpression and gene amplification in squamous cell carcinomas of the esophagus Int J Cancer 2006 118 1173 1180 10.1002/ijc.21454 16161046

[B16] Ohashi K Sequist LV Arcila ME Moran T Chmielecki J Lin YL Pan Y Wang L de Stanchina E Shien K Aoe K Toyooka S Kiura K Fernandez-Cuesta L Fidias P Yang JC Miller VA Riely GJ Kris MG Engelman JA Vnencak-Jones CL Dias-Santagata D Ladanyi M Pao W Lung cancers with acquired resistance to EGFR inhibitors occasionally harbor BRAF gene mutations but lack mutations in KRAS, NRAS, or MEK1 Proc Natl Acad Sci U S A 2012 109 31 E2127 E2133 10.1073/pnas.1203530109 22773810 PMC3411967

[B17] Radojicic J Zaravinos A Spandidos DA HPV, KRAS mutations, alcohol consumption and tobacco smoking effects on esophageal squamous-cell carcinoma carcinogenesis Int J Biol Markers 2012 27 1 1 12 10.5301/JBM.2011.8737 22020370

[B18] Lyronis ID Baritaki S Bizakis I Krambovitis E Spandidos DA K-ras mutation, HPV infection and smoking or alcohol abuse positively correlate with esophageal squamous carcinoma Pathol Oncol Res 2008 14 267 273 10.1007/s12253-008-9032-1 18592405

[B19] Marx AH Zielinski M Kowitz CM Dancau AM Thieltges S Simon R Choschzick M Yekebas E Kaifi JT Mirlacher M Atanackovic D Brümmendorf TH Fiedler W Bokemeyer C Izbicki JR Sauter G Homogeneous EGFR amplification defines a subset of aggressive Barrett’s adenocarcinomas with poor prognosis Histopathology 2010 57 3 418 426 10.1111/j.1365-2559.2010.03643.x 20840671

[B20] Tasioudi KE Saetta AA Sakellariou S Levidou G Michalopoulos NV Theodorou D Patsouris E Korkolopoulou P pERK activation in esophageal carcinomas: clinicopathological associations Pathol Res Pract 2012 208 7 398 404 10.1016/j.prp.2012.05.009 22658382

[B21] Lord RV O’Grady R Sheehan C Field AF Ward RL K-ras codon 12 mutations in Barrett’s oesophagus and adenocarcinomas of the oesophagus and oesophagogastric junction J Gastroenterol Hepatol 2000 15 730 736 10.1046/j.1440-1746.2000.02163.x 10937677

[B22] Akagi I Miyashita M Makino H Nomura T Hagiwara N Takahashi K Cho K Mishima T Ishibashi O Ushijima T Takizawa T Tajiri T Overexpression of PIK3CA is associated with lymph node metastasis in esophageal squamous cell carcinoma Int J Oncol 2009 34 3 767 775 19212681 10.3892/ijo_00000202

[B23] Maeng CH Lee J van Hummelen P Park SH Palescandolo E Jang J Park HY Kang SY MacConaill L Kim KM Shim YM High-throughput genotyping in metastatic esophageal squamous cell carcinoma identifies phosphoinositide- 3-kinase and BRAF mutations PLoS One 2012 7 8 e41655 10.1371/journal.pone.0041655 22870241 PMC3411721

[B24] Phillips WA Russell SE Ciavarella ML Choong DY Montgomery KG Smith K Pearson RB Thomas RJ Campbell IG Mutation analysis of PIK3CA and PIK3CB in esophageal cancer and Barrett’s esophagus Int J Cancer 2006 118 10 2644 2646 10.1002/ijc.21706 16380997

[B25] Mori R Ishiguro H Kimura M Mitsui A Sasaki H Tomoda K Mori Y Ogawa R Katada T Kawano O Harada K Fujii Y Kuwabara Y PIK3CA mutation status in Japanese esophageal squamous cell carcinoma J Surg Res 2008 145 2 320 326 10.1016/j.jss.2007.03.044 18262558

[B26] Hou GQ Lu ZM Liu MY Liu HM Xue LX Mutational analysis of the PTEN gene and its effects in esophageal squamous cell carcinoma Dig Dis Sci 2011 56 5 1315 1322 10.1007/s10620-010-1474-0 21116717

[B27] Ma J Zhang J Ning T Chen Z Xu C Association of genetic polymorphisms in MDM2, PTEN and P53 with risk of esophageal squamous cell carcinoma J Hum Genet 2012 57 4 261 264 10.1038/jhg.2012.15 22336889

[B28] Hu YC Lam KY Tang JC Srivastava G Mutational analysis of the PTEN/MMAC1 gene in primary oesophageal squamous cell carcinomas Mol Pathol 1999 52 6 353 356 10.1136/mp.52.6.353 10748870 PMC395722

[B29] Kato H Arao T Matsumoto K Fujita Y Kimura H Hayashi H Nishiki K Iwama M Shiraishi O Yasuda A Shinkai M Imano M Imamoto H Yasuda T Okuno K Shiozaki H Nishio K Gene amplification of EGFR, HER2, FGFR2 and MET in esophageal squamous cell carcinoma Int J Oncol 2013 42 4 1151 1158 23426935 10.3892/ijo.2013.1830 PMC3622677

